# The beneficial impact of a low-carbohydrate diet on glycemic variability in insulin-deficient diabetes

**DOI:** 10.3389/fnut.2025.1733037

**Published:** 2026-01-09

**Authors:** Jing Song, Jingjie Wang, Jing Wan, Ye Lu, Wenjie Wang, Li Li, Jun Wang, Li Wei, Jun Yin

**Affiliations:** 1 Department of Endocrinology and Metabolism, Shanghai Eighth People’s Hospital, Shanghai, China; 2 Department of Endocrinology and Metabolism, Shanghai Sixth People’s Hospital Affiliated to Shanghai Jiao Tong University School of Medicine, Shanghai Clinical Center for Diabetes, Shanghai Diabetes Institute, Shanghai Key Laboratory of Diabetes Mellitus, Shanghai, China; 3 Department of Nutrition, Shanghai Eighth People’s Hospital, Shanghai, China

**Keywords:** continuous glucose monitoring, C-peptide, glycemic variability, ketone body, low-carbohydrate diet

## Abstract

**Aims:**

To evaluate the short-term metabolic effects of a low-carbohydrate diet (LCD) compared with a control diet (CON) in hospitalized patients with insulin-deficient diabetes, with particular focus on glycemic variability (GV).

**Methods:**

This non-randomized clinical trial included 359 inpatients with fasting C-peptide levels ≤1.0 ng/mL. Participants chose either an LCD or CON during hospitalization. GV was assessed using capillary and continuous glucose monitoring, with coefficient of variation (CV) as primary outcome. Secondary measures included other GV metrics, antidiabetic medication use, ketone levels, and adverse events. Subgroup analysis was conducted based on baseline C-peptide levels.

**Results:**

LCD led to a greater reduction in CV (diet-by-time interaction, *p* = 0.03), as well as improvements in MAGE, SD, TIR, and iAUC compared with CON. LCD patients also showed reduced requirements for oral agents and insulin injection at discharge. Benefits were more pronounced in individuals with lower C-peptide levels (P for interaction = 0.002). No increase in adverse events was observed. Ketone levels increased in the LCD group and negatively correlated with discharge blood glucose and CV.

**Conclusion:**

Short-term LCD intervention improved GV in insulin-deficient diabetes, especially in patients with more significant *β*-cell dysfunction, with blood ketones negatively correlated with blood glucose and GV.

**Clinical trial registration:**

ChiCTR2000038006, https://www.chictr.org.cn/showproj.html?proj=60712

## 1 Introduction

Diabetes mellitus (DM) is increasingly recognized as a heterogeneous metabolic condition with varying degrees of insulin resistance and *β*-cell dysfunction (1). Among its subtypes, endogenous-insulin-deficient diabetes, which is characterized by markedly reduced endogenous insulin secretion and low fasting C-peptide levels, represents a clinically distinct and metabolically vulnerable subgroup. These patients usually rely on exogenous insulin to maintain glycemic control, which indicates substantial challenges in clinical practice. Precise insulin dosing typically requires delicate carbohydrate counting and insulin sensitivity calculations. However, under common dietary patterns, it is hard to accurately estimate carbohydrate intake (2). Moreover, high carbohydrate consumption necessitates larger insulin injection doses, which can lead to increased variability in subcutaneous insulin absorption (3). Patients receiving intensive insulin regimens are also subjected to frequent injections at limited anatomical sites, which increases the risk of local lipohypertrophy and further impairs insulin absorption (4). These factors all contribute to the complexity of glycemic management in patients with insulin-deficient diabetes.

Glycemic variability (GV), referring to the degree of fluctuation between glucose peaks and nadirs, has emerged as a clinically relevant metric that complements traditional markers, such as HbA1c, in assessing glycemic control (5, 6). As one of the three key components of the “glycemic triumvirate,” GV has been increasingly linked to diabetic complications (7, 8). In patients with insulin-deficient diabetes, GV is often more pronounced and challenging to manage due to reduced endogenous insulin buffering and high dependence on exogenous insulin. Therefore, it is important to regulate GV as a therapeutic target of DM, particularly in this high-risk population.

Dietary intervention remains a cornerstone of DM management, and among various approaches, low-carbohydrate diets (LCDs) have attracted clinical attention since the early 20th century as a means to regulate glycemia (9). LCDs are commonly defined as those in which carbohydrate intake constitutes less than 130 grams per day (10). Such restriction of carbohydrate intake, as supported by the consensus of the American Diabetes Association (ADA) (11), has been shown to exert a beneficial effect on lowering HbA1c levels, decreasing the requirement for antidiabetic medications, and maintaining glucose homeostasis (3, 12, 13). Notably, most of these findings are primarily derived from studies involving individuals with obesity and/or type 2 diabetes, who often manifest as hyperinsulinemia and insulin resistance (14, 15). In contrast, data on the efficacy and safety of LCD in patients with insulin-deficient diabetes remain scarce, especially regarding the impact of LCD on GV. Moreover, although residual *β*-cell function is known to influence glucose stability, whether markers such as C-peptide can identify subgroups that respond differently to carbohydrate restriction has not been systematically investigated. Addressing these gaps is clinically relevant, as insulin-deficient patients often experience pronounced GV and may exhibit heterogeneous metabolic responses to dietary modification.

Results from our pilot study demonstrate that a low-carbohydrate diet significantly reduced glycemic variability and insulin requirements without increasing the risk of hypoglycemia or ketoacidosis in patients with insulin-deficient diabetes (16). However, the pilot study was limited by its small sample size (n = 44), and the broader clinical relevance of its findings required further confirmation. To address these gaps, we conducted a larger non-randomized clinical trial to evaluate the short-term effects of an LCD compared to a low-fat control (CON) diet on GV in hospitalized patients with endogenous-insulin-deficient diabetes. GV was comprehensively assessed using multiple validated metrics, including coefficient of variation (CV), mean amplitude of glycemic excursions (MAGE), time in range (TIR), etc. C-peptide levels were further evaluated as a potential modifier of dietary response, and alterations in blood ketone levels were analyzed to ensure the safety of LCDs in insulin-deficient individuals and explore their possible role in glycemic regulation. Findings from this study may contribute to the development of more personalized dietary strategies for diabetes management, particularly in a subgroup of patients with severely impaired insulin secretion.

## 2 Materials and methods

### 2.1 Study population

The study recruited 359 subjects with endogenous-insulin-deficient diabetes from patients hospitalized at the Department of Endocrinology and Metabolism, Xuhui Branch of Shanghai Sixth People’s Hospital, from February 2021 to November 2023. The inclusion criteria were as follows:(1) a diagnosis of diabetes mellitus by the 2010 ADA criteria; and (2) fasting C-peptide level ≤1.0 ng/mL, indicating markedly endogenous insulin deficiency. Patients with any of the following conditions were excluded: (1) pregnancy or lactation, (2) episodes of acute diabetic complications within 1 month before enrollment, (3) severe liver or renal dysfunction, (4) severe cardiovascular disease or peripheral arterial disease, (5) any febrile or infectious illness, (6) history of malignancy or psychiatric disorders, (7) history of alcohol or drug abuse and (8) Poor adherence to study protocol.

This study was approved by the Institutional Review Board of Shanghai Eighth People’s Hospital. The trial was registered at www.chictr.org.cn with clinical trial registration number ChiCTR2000038006. All participants provided written informed consent before enrollment.

### 2.2 Design and data collection

Participants were assigned to the LCD or CON group based on their willingness to follow a carbohydrate-restricted diet at the time of admission. Patients who agreed to adopt LCD were placed in the LCD group, whereas those who declined were assigned to the CON group. After group assignment, all meals were provided throughout hospitalization via the hospital’s standardized food delivery system according to the assigned diet. The CON comprised approximately 45% carbohydrates, 39% fat, and 16% protein, while the LCD comprised approximately 15% carbohydrates, 69% fat, and 16% protein. The total daily caloric intake of each participant was individually calculated based on ideal body weight and daily physical activity intensity. Ideal body weight(kg) was defined as height(cm) minus 105, and caloric intake was set at 25 kcal/kg per day based on a uniform classification of light physical activity intensity during hospitalization. Median intervention durations were 9 (IQR 7–11) days in the CON group and 9 (IQR 8–10) days in the LCD group. Prior to the intervention, all participants received education on their respective diets to promote adherence.

Baseline demographic data, medical history, laboratory test results, and medication information were extracted from the hospital’s electronic medical record system. Clinical variables with potential relevance to glycemic control were collected comprehensively. Body mass index (BMI) was calculated as weight (kg) divided by the square of height (m^2^). Blood pressure was measured three times at 5-min intervals on the right arm in a seated position, and the average of the three measurements was used in the analysis. Absolute change in insulin dose was calculated as the difference between discharge and admission doses. The percent change of insulin dosage was computed among participants who were using insulin at admission, defined as (discharge dose − admission dose) / admission dose × 100%. Patients with a baseline insulin dose of 0 U were not included in the percent-change analysis as percent change is not mathematically defined in this context.

Capillary blood glucose measurements were uniformly performed by ward nurses at nine scheduled time points daily (06:30, 09:00, 10:30, 13:00, 16:30, 19:00, 21:00, 00:00, and 03:00) using the Accu-Chek Performa Blood Glucose Meters (Roche, Switzerland). These data were used to calculate glycemic control and GV metrics, including mean blood glucose, standard deviation (SD), CV, MAGE, TIR (time between 3.9 and 10.0 mmol/L), time above range (>10.0 mmol/L, TAR), and time below range (time <3.9 mmol/L, TBR). Additionally, 99 participants were equipped with a blinded continuous glucose monitoring (CGM) device (iPro2 Recorder, Medtronic, Ireland) inserted into the subcutaneous tissue of their upper arm. Interstitial glucose readings were collected by the device every 5 min throughout hospitalization. CGM data were included if the exported file contained > 90% of data points 24 h after admission and 24 h before discharge. Individual incremental area under the curve (iAUC) values were calculated using the trapezoidal method. Group comparisons of iAUC were performed using independent samples t-tests through GraphPad Prism 10.

Blood ketone levels of participants in the LCD group were measured on a daily basis using the FreeStyle Optium Neo H device (Abbott, UK) at four scheduled time points (06:30, 10:30, 16:30, and 21:00) during intervention. The mean of these four values was calculated as the daily average blood ketone level. Data on adverse events, including diabetic ketoacidosis (DKA) and hypoglycemia (defined as glucose <3.9 mmol/L), were systematically recorded throughout the intervention period. Discharge prescriptions for antidiabetic medications were also documented for all participants.

### 2.3 Statistical analysis

The sample size of this study was estimated based on the primary endpoint of glycemic variability, expressed as CV. In our previous pilot study of 44 matched patients with insulin-deficient diabetes, a LCD reduced CV by approximately 7–8% compared with CON, with a SD of 8–12% (16). To avoid overestimation and ensure a conservative design, we assumed a between-group difference of 3.3% with an SD of 10% for the present study. Based on these assumptions, a minimum of 146 participants per group (total n = 292) would be required to achieve 80% power at a two-sided *α* of 0.05. Considering the non-randomized design, potential imbalance in group sizes, and possible data loss, we planned to recruit approximately 360 participants. Eventually, 359 subjects were enrolled.

Statistical analyses were conducted using the R software, version 4.4.1. Data normality was assessed using the Shapiro–Wilk test and quantile-quantile (Q-Q) plots. Statistical evaluations of baseline characteristics and medication use between groups were conducted through independent samples t-tests when the variable was normally distributed or the Mann–Whitney U test when a nonnormality was suggested. Chi-square tests and Fisher’s exact test were conducted to compare the frequencies of categorical variables. Owing to the inpatient setting of this study, the dataset exhibited a high level of completeness, with less than 5% of values missing. Accordingly, all analyses were performed on the available data.

The primary objective was to test the superiority of an LCD compared with a CON for improving GV after short-term dietary intervention. Thus, a linear mixed model was applied with fixed effects for timepoints (Admission compared with Discharge), dietary intervention group, the interaction between timepoint and dietary intervention group, and a random effect for each participant. Estimates of Relative LCD Effect on CV Across Intervention with 95% CI are reported as the main analyses of interest. Other metrics of GV and glycemic control were analyzed similarly as secondary outcomes.

Simple and stepwise multiple linear regressions were applied to identify potential factors significantly associated with stable glycemic control (discharge CV < 36% (17)). A stratified analysis based on the selected factor C-peptide was further performed using a Poisson regression model with a log link to estimate relative risks (RRs) of achieving discharge CV < 36%. Participants were classified using a fasting C-peptide threshold of 0.5 ng/mL, a cutoff commonly used in clinical research to indicate markedly reduced *β*-cell reserve and insulin deficiency (18–20). An interaction term between dietary group and C-peptide subgroup was included in the model to evaluate potential effect modification.

Propensity score matching (PSM) was applied to CON and LCD participants at a 1:1 ratio, using age, type 2 DM (T2DM) duration, and systolic blood pressure (SBP) as matching variables. The caliper value was set to 0.2. Analyses based on the matched cohort were similar in methodology to the main analyses.

Paired *t*-tests were used to assess within-group changes in average blood ketone levels from admission to discharge in each dietary group. To further examine the association between ketone metabolism and blood glucose regulation under the LCD intervention, an LMM was conducted using multiple paired blood ketone and glucose values collected at four standardized timepoints (06:30, 10:30, 16:30, and 21:00) during the final 24 h before discharge among LCD participants. In this model, blood glucose level was specified as the dependent variable, with corresponding blood ketone level and timepoint included as fixed effects, and participant ID included as a random effect to account for repeated measurements. In addition, a simple linear regression model was performed in the LCD group to examine the association between ketone metabolism and GV, with discharge CV as the dependent variable and discharge average ketone level as the independent variable. Chi-square tests were used to compare the incidence of adverse events, including DKA and hypoglycemia, between the two groups. All statistical tests conducted in this study were two-tailed, with statistical significance set at a *p* < 0.05.

## 3 Results

### 3.1 Baseline characteristics of participants by diet group

**Figure 1:**
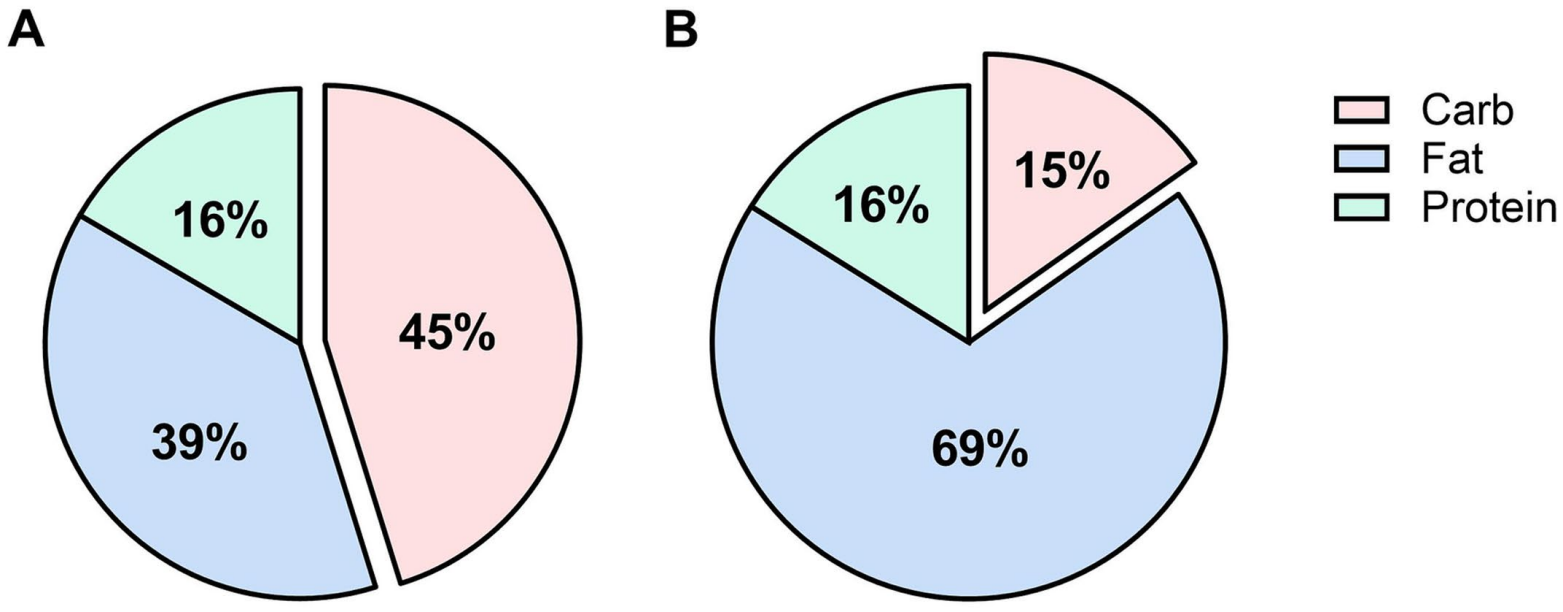
Macronutrient composition of diets. Macronutrient composition of diets during the intervention period for the CON **(A)** and LCD **(B)** groups. CON, low-fat control diet; LCD, low-carbohydrate diet.

A total of 359 patients with endogenous insulin-deficient diabetes who met the inclusion criteria were enrolled in this study. Among which, 225 patients opted for a standard control diet, while 134 chose an LCD (Figure 1). Comparison of the baseline characteristics between the two diet groups revealed that patients in the LCD group were younger and had a shorter disease duration, indicating that the LCD is usually preferred by the younger population. In addition, patients of the CON group exhibited significantly higher SBP compared to those in the LCD group (*p* < 0.05). Notably, no other significant differences were observed between the two diet groups (Table 1).

**Table 1:** Baseline characteristics of study participants.^1.^

Characteristics	CON	LCD	Statistics	*p*-value
	*n* = 225	*n* = 134		
Age, years	69 [63–76]	59.5 [50–67.75]	8.0	<0.01
Male Gender, *n* (%)	124 (55.1%)	66 (49.3%)	0.9	0.3
Duration, years	15 [8.75–20]	7.5 [1–15]	5.8	<0.01
SBP, mmHg	134.9 ± 19.0	129.8 ± 17.7	2.5	<0.01
DBP, mmHg	76.2 ± 9.4	77.6 ± 10.0	−1.4	0.2
BMI, kg/m2	23.1 [20.8–24.9]	22.51 [20.1–24.4]	1.6	0.1
C-peptide, ng/ml	0.58 [0.38–0.81]	0.58 [0.26–0.8]	0.9	0.4
HbA1c, %	10.3 [8.5–12.2]	10.4 [8.5–12.5]	−0.4	0.7
GA, %	32.1 ± 9.7	31.7 ± 9.4	0.5	0.7

^1^Data are expressed as mean ± SD, median (interquartile range) or n (%). CON, low-fat control diet group; LCD, low-carbohydrate diet group, SBP, systolic blood pressure; DBP, diastolic blood pressure; BMI, body mass index; HbA1c, glycated hemoglobin A1c; GA, glycated albumin.

### 3.2 CV and other metrics of glycemic variability

Table 2 summarizes the effect of dietary intervention on blood glucose variability, namely the primary outcome and secondary outcomes of this study, based on a linear mixed model incorporating a “diet-by-time” interaction term. The primary outcome, CV, showed a greater reduction in the LCD group compared to the CON group from admission to discharge. Specifically, the mean (±SD) value of CV decreased from 35.4 ± 12.6% to 21.9 ± 8.4% in the LCD group and from 37 ± 12% to 26.8 ± 9% in the CON group. The relative effect of the LCD on CV was −3.4% (95% CI: −6.4 to −0.3, *p* = 0.03), indicating a significant diet-by-time interaction favoring the LCD. As an additional sensitivity analysis, an analysis of covariance (ANCOVA) adjusting for baseline CV was performed (Supplementary Table S6), and the results were consistent with the primary analysis.

**Table 2:** Comparison of dietary effects on improvements in glycemic variability across the intervention period.^1^

Variables	Admission		Discharge		Relative LC effect across intervention	*P*-value^2^
	CON	LCD	CON	LCD		
CV (%)	37 ± 12	35.4 ± 12.6	26.8 ± 9	21.9 ± 8.4	−3.4 (−6.4 to −0.3)	0.03
MAGE	7.6 ± 3.8	7.3 ± 3.8	4.8 ± 2.3	3.4 ± 1.8	−1.1 (−2.0 to −0.3)	<0.01
SD	4 ± 1.6	3.8 ± 1.6	2.4 ± 1.1	1.8 ± 0.8	−0.4 (−0.8 to −0.1)	0.02
Mean	10.9 ± 2.6	10.7 ± 2.5	8.8 ± 1.7	8.1 ± 1.4	−0.6 (−1.2 to 0.02)	0.06
TIR (%)^3^	50.5 ± 24.4	53 ± 26.3	72.9 ± 22	82 ± 21.7	0.6 (0.2 to 0.9)	<0.01
TAR (%)^3^	47.3 ± 24.5	45.3 ± 26.9	25.7 ± 21.3	16.9 ± 21.3	−0.6 (−1.0 to −0.3)	<0.01
TBR (%)^3^	2.2 ± 8.1	1.7 ± 5.1	1.4 ± 7.3	1.1 ± 3.9	0.01 (−0.3 to 0.3)	0.9

^1^Data by group are presented as mean ± SD, relative effect of LC diet across intervention is presented as effect estimates (95% CI). ^2^Data analyzed via linear mixed model with fixed effects for timepoints (Admission compared with Discharge), dietary intervention group, the interaction between timepoint and dietary intervention group, and a random effect for each participant. ^3^Data were converted to and are presented as proportion of time in range and analyzed via mixed effects beta model. Interpret effect estimates as % relative change in odds. CON, low-fat control diet group; LCD, low-carbohydrate diet group; CV, coefficient of variation; MAGE, mean amplitude of glycemic excursions; SD, standard deviation; TIR, time between 3.9 and 10 mmol/L; TAR, time above 10 mmol/L; TBR, time below 3.9 mmol/L.

Several other metrics of glycemic control and GV were assessed as secondary outcomes, including mean glucose levels, MAGE, SD, TIR, TAR, and TBR. MAGE and SD were significantly lower at discharge in the LCD group than in the CON group, with a relative LCD effect of −1.1 (*p* < 0.01) for MAGE and −0.4 (*p* = 0.02) for SD. TIR improved in both groups, but the LCD group showed a significantly higher increase, as indicated by the relative effect (p < 0.01). Similarly, TAR decreased more substantially in the LCD group (p < 0.01). Mean glucose levels showed a greater decreasing trend in the LCD group, although the difference between two diet groups was not statistically significant (*p* = 0.06). No significant difference was observed between groups for TBR (*p* = 0.9).

**Figure 2:**
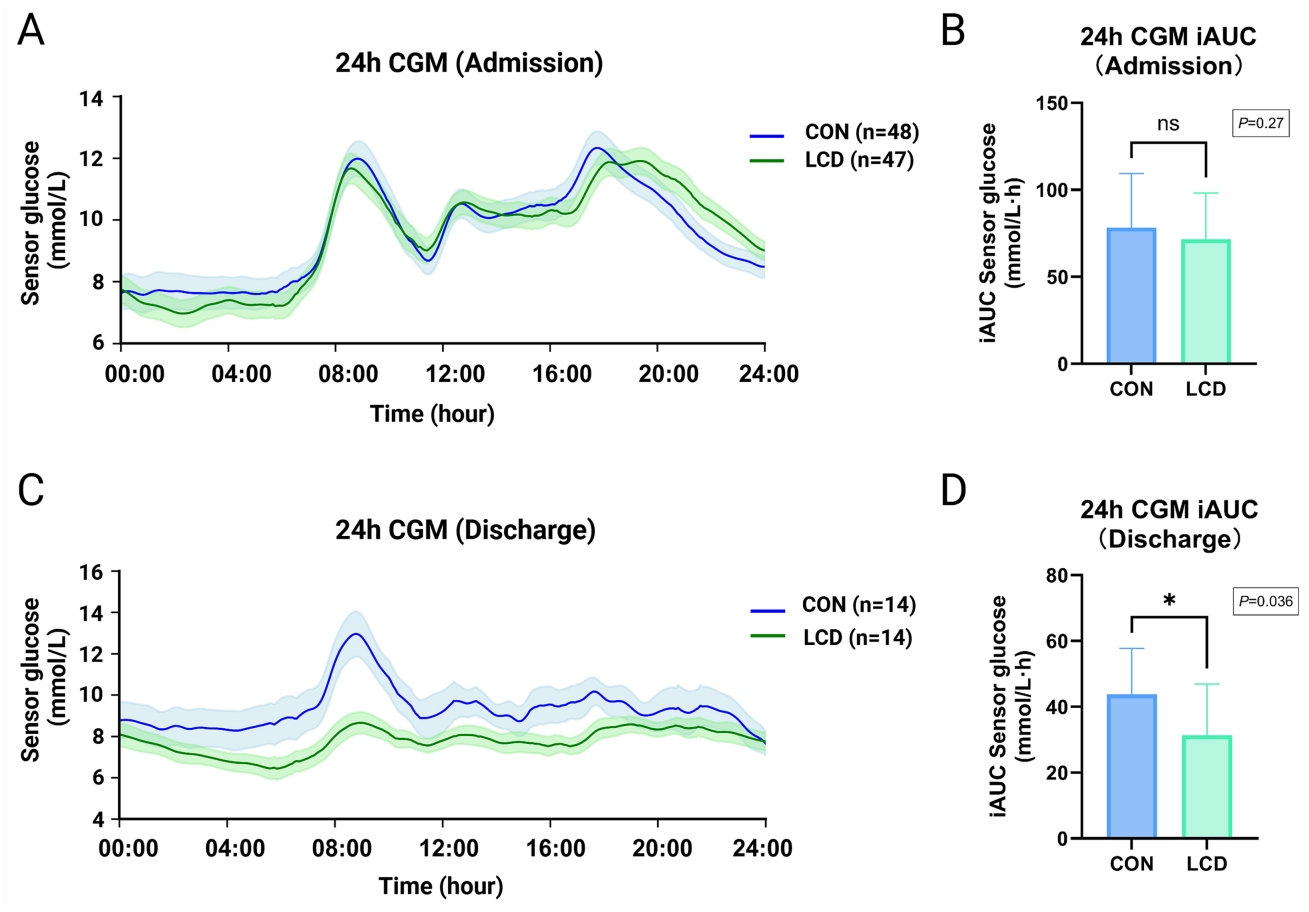
Comparisons of 24-h sensor glucose levels and glycaemic variability between different dietary intervention at admission and discharge. **(A,B)** 24-h sensor glucose trend curve at admission and corresponding comparisons of curve iAUC between two diet groups. **(C,D)** 24-h sensor glucose trend curve at discharge and corresponding comparisons of curve iAUC between two diet groups. Values are presented as means ± SEM for the curves and means ± SD for iAUC (mmol/L·h). Statistical comparisons were performed using independent samples *t*-tests, with *p* values displayed in text boxes. ns, Not significant; * *p* < 0.05 from CON. CGM, Continuous glucose monitoring; iAUC, incremental AUC; CON, low-fat control diet; LCD, low-carbohydrate diet.

Additionally, 24-h sensor glucose profiles and iAUC values at admission and discharge are presented in Figure 2. Among patients who underwent CGM monitoring, only a subset had analyzable discharge-day data (14 in each group). This was because only a limited number of CGM devices were available in the inpatient unit and the sensors had to be rotated among all hospitalized patients for routine clinical use, which should be taken into account when interpreting the CGM-derived outcomes. At admission, glucose trends and iAUC values were similar between the two groups (*p* = 0.27). However, by discharge, the LCD group exhibited a significantly lower iAUC compared to the CON group (*p* = 0.036), reflecting reduced postprandial and overall glucose exposure throughout the day. Although iAUC is not a direct measure of GV, this observed trend difference and lower iAUC in the LCD group at discharge are consistent with improvements in glycemic control and GV, as reported in Table 2.

### 3.3 Analysis of antidiabetic medication use

Table 3 presents the comparison of antidiabetic medication use between the two diet groups. At baseline, the mean daily insulin dose was significantly lower in the LCD group (17.6 ± 13.1 units) than in the CON group (22.0 ± 14.6 units, *p* = 0.004). This difference became even more pronounced (13.9 ± 8.0 vs. 25.5 ± 13.6 units, *p* < 0.001) at discharge. Among participants receiving insulin at admission, percent change in insulin dose was also examined and showed a similar between-group pattern (Supplementary Table S7). Importantly, the frequency of daily insulin injections at discharge was also significantly reduced in the LCD group compared with the CON group, indicating that LCD patients not only required lower doses but also fewer injections per day (1 (1) vs. 2 (1–4) times, *p* < 0.001). Regarding oral antidiabetic agents, the number of drug classes used per patient (0/1/2/3 classes) was comparable between the two groups at admission (*p* = 0.11). However, by discharge, significantly fewer patients in the LCD group required multiple oral agents, and a larger proportion were managed without any oral medications (p < 0.001). Such results suggest that the LCD successfully reduced not only insulin dosage but also the overall intensity of insulin therapy, as reflected by injection frequency, as well as the need for oral antidiabetic medications.

**Table 3:** Comparison of daily insulin dosage and oral anti-diabetic agents between different dietery intervention at admission and discharge.

Variables	CON	LCD	*P*-value
Daily insulin dosage^1^			
Admission	22.0 ± 14.6	17.6 ± 13.1	0.004
Discharge	25.5 ± 13.6	13.9 ± 8.0	<0.001
Dosage difference	3.5 ± 17.8	−3.7 ± 14.2	<0.001
Insulin injection frequency at discharge (per day)^2^	2 [1–4]	1 [1–1]	<0.001
Types of oral anti-diabetic agents^3^ (0/1/2/3 types)			
Admission	(176/41/7/1)	(117/16/1/0)	0.11
Discharge	(42/96/82/5)	(98/32/4/0)	<0.001

^1^Data are expressed as mean ± SD (*p*-value from independent samples *t*-test). ^2^Data are expressed as median (interquartile range) (*p*-value from Mann–Whitney U test). ^3^Data are expressed as (n0/n1/n2/n3) (*p-*value from Fisher’s exact test). CON, low-fat control diet group; LCD, low-carbohydrate diet *group*.

### 3.4 Stratified analysis by C-peptide level based on regression findings

To explore whether factors other than the diet group were associated with GV, linear regression analyses were performed with discharge CV as the dependent variable (Table 4). The results from simple linear regression analysis demonstrated that diet group, BMI, C-peptide, DBP, and admission CV had a significant correlation with discharge CV. These variables were subsequently included in a stepwise multiple regression model. The final model retained three independent variables: dietary group, BMI, and C-peptide, all of which remained significantly associated with discharge CV (*p* < 0.001). Specifically, the LCD, higher BMI, and higher C-peptide levels were independently associated with lower CV at discharge. The model explained 16.9% of the variance in discharge CV.

**Table 4:** Linear regression analysis evaluating discharge CV as dependent variable.

Independent variables	Simple regression				Stepwise multiple regression						
	Unstandardized coefficient	Standardized β coefficient	*t*-value	*p*-value	Unstandardized coefficient	Standardized β coefficient	*t*-value	*p*-value	Relative Weight	Unadjusted R^2^ (%)	Adjusted R^2^ (%)
Diet (CON / LCD)	−4.94	−0.54	−5.16	<0.001	−5.41	−0.6	−5.97	<0.001	0.08	17.6	16.9
Age, years	0.03	0.05	0.86	0.39							
Gender	−1.04	−0.11	−1.08	0.28							
Duration	0.04	0.04	0.73	0.46							
SBP	−0.04	−0.08	−1.52	0.13							
DBP	−0.12	−0.12	−2.35	0.02							
BMI	−0.62	−0.21	−4.05	<0.001	−0.5	−0.17	−3.41	<0.001	0.04		
C-peptide	−8.4	−0.26	−5.19	<0.001	−7.66	−0.24	−4.86	<0.001	0.06		
HbA1c	0.10	0.04	0.79	0.43							
GA	0.10	0.10	1.91	0.06							
Admission CV	0.11	0.15	2.79	0.006							

CON, low-fat control diet group; LCD, low-carbohydrate diet group, SBP, systolic blood pressure; DBP, diastolic blood pressure; BMI, body mass index; HbA1c, glycated hemoglobin A1c; GA, glycated albumin; CV, coefficient of variation.

Given these findings, both BMI and C-peptide emerged as important factors that may modify the effect of dietary intervention. However, due to the limited number of participants with obesity (BMI > 28) in our cohort, subgroup analysis based on BMI was not feasible. Therefore, we conducted a stratified analysis based on baseline C-peptide levels to investigate further the potential interaction between C-peptide levels and dietary intervention (Table 5). In participants with a lower C-peptide level (<0.5 ng/mL), the proportion achieving stable glycemic control (discharge CV < 36% (17)) was significantly higher in the LCD group than in the CON group. In contrast, no difference was observed between different diet groups among participants with a higher C-peptide level (≥0.5 ng/mL). A formal test for interaction confirmed the significant diet-by-C-peptide interaction effect on discharge CV (P for interaction = 0.002), suggesting that patients with lower endogenous insulin secretion may benefit more from a low-carbohydrate dietary intervention in terms of GV improvement.

**Table 5:** Dietary intervention and low discharge CV (discharge CV < 36%) in all participants, stratified by C-peptide levels.^1.^

C-peptide levels	CON		LCD		RR (95 CI%)	*p-*value
	*n*	Discharge cv < 36%, n (%)	*n*	Discharge cv < 36%, n (%)		
< 0.5 ng/ml	97	70 (72.2%)	56	52 (92.9%)	1.3 (1.1–1.5)	< 0.001
≥ 0.5 ng/ml	128	120 (93.8%)	78	73 (93.6%)	1.0 (0.93–1.07)	0.97
Test for interaction^2^						0.002

^1^Data analyzed via generalized linear model adjusted for baseline glycaemic variability (admission CV). ^2^*P*-value for interaction test: 2-way interaction of diet (Low-Carbohydrate vs. Normal Diet) and C-peptide levels (<0.5 vs. >0.5) on discharge CV. CON, low-fat control diet group; LCD, low-carbohydrate diet group; RR, relative risk; CI, confidence interval.

### 3.5 Sensitivity analysis using propensity score–matched cohort

Given the baseline imbalances in age, disease duration, and SBP observed between the two diet groups in the primary analysis, a sensitivity analysis was performed using a propensity score–matched cohort to minimize potential confounding. Participants in the LCD and CON groups were matched 1:1 based on age, disease duration, and SBP, yielding two groups with balanced baseline characteristics (Supplementary Table S1).

The results from the matched cohort closely mirrored those of the primary analysis. As shown in Supplementary Table S2, the LCD group exhibited significantly greater improvements in GV upon discharge, including CV, MAGE, SD, and TIR, with effect sizes and statistical significance similar to those in the unmatched analysis. Supplementary Table S3 showed that the LCD group continued to have a significantly greater reduction in insulin dosage and used fewer types of oral antidiabetic medications at discharge. Stratified analysis by C-peptide (Supplementary Table S4) replicated the significant interaction observed in the unmatched analysis, indicating that patients with lower C-peptide levels derived greater benefit from the LCD. Collectively, these sensitivity analyses in a baseline-balanced cohort confirmed the robustness and consistency of the primary results.

### 3.6 Blood ketone body levels, adverse events, and safety analysis

In this study, no cases of DKA were reported in either group during hospitalization, indicating that short-term intervention of the LCD was safe in this inpatient setting. The incidence of hypoglycemia was also similar between groups. A total of 45 participants (20.0%) in the CON group and 24 participants (17.9%) in the LCD group experienced at least one episode of hypoglycemia, with no statistically significant difference between the two diet groups (χ^2^ = 0.12, *p* = 0.73). These findings suggest that a short-term LCD does not increase the risk of DKA and may even reduce the frequency and duration of hypoglycemic episodes in patients with diabetes.

**Figure 3:**
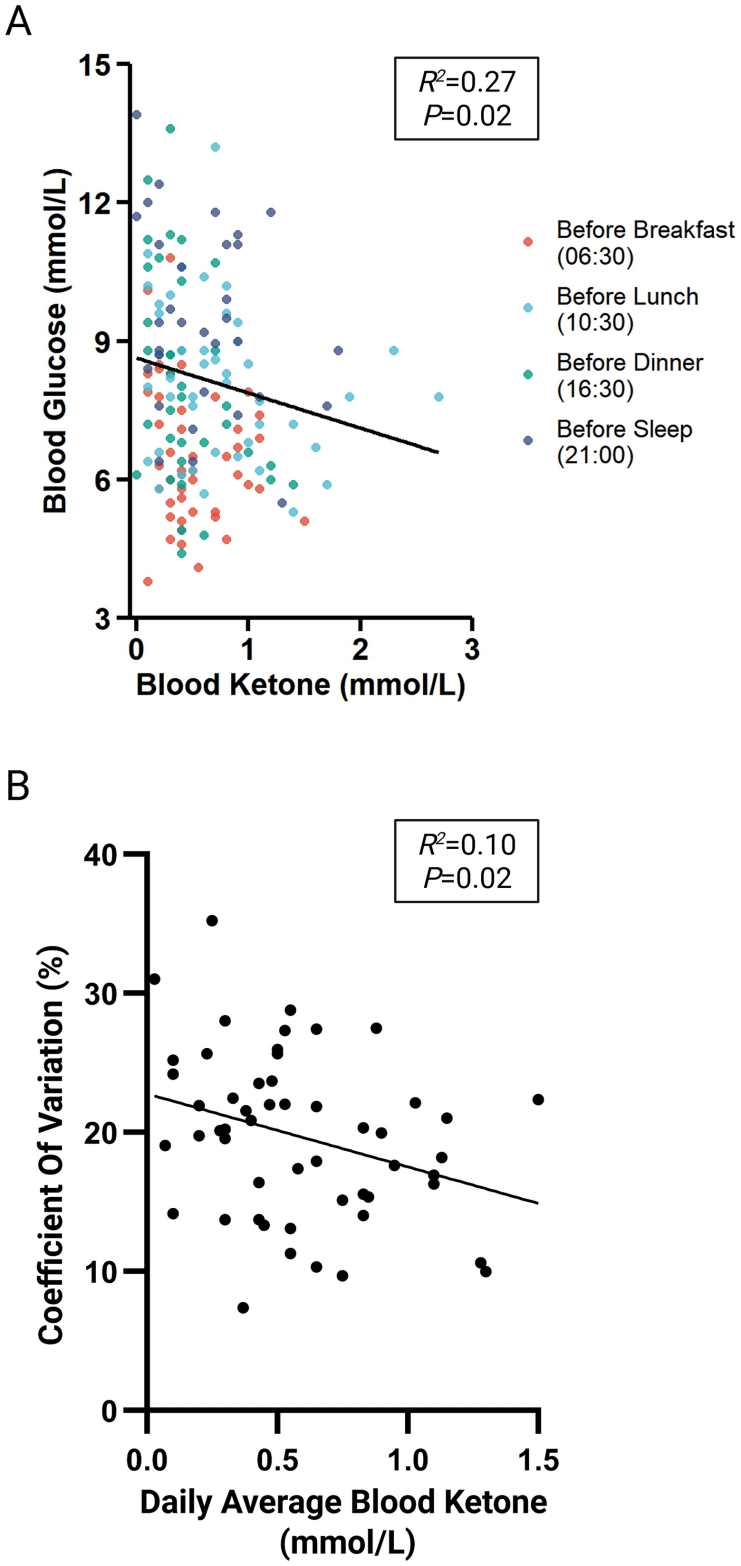
Associations of blood ketone levels with blood glucose and GV in the LCD group at discharge. **(A)** Association between blood ketone levels and blood glucose in the LCD group at discharge. R^2^ and *p-*value are from LMM analyses. Dot colors correspond to four standardized timepoints (06:30, 10:30, 16:30, and 21:00), and the solid black trend line indicates the main effect of blood ketone predicted by the model while controlling for individual variability and timepoint (*n* = 53). **(B)** Associations between mean fasting blood ketone and CV in the LCD group at discharge. R^2^ and *p*-value are from simple linear regression analyses (*n* = 53).

To further investigate the metabolic effects of the LCD, changes in daily average blood ketone levels were analyzed. As shown in Table 6, ketone levels at discharge showed no significant change relative to admission in the CON group, while a substantial increase was observed in the LCD group (*p* < 0.001). Subsequently, a LMM model was applied in the LCD group to assess the association between blood ketone concentration and glucose level during the final 24 h before discharge, based on repeated measurements across four standardized timepoints. The model revealed a robust inverse association (Figure 3, R^2^ = 0.27, *p* = 0.02), indicating that higher ketone levels were generally associated with lower glucose levels after adjusting for timepoint and individual variation. Moreover, in the LCD group, average ketone levels at discharge were negatively correlated with CV (Figure 3, R^2^ = 0.10, p = 0.02), suggesting a possible association between ketone metabolism and GV.

**Table 6:** Impact of reference diet and LC diet on blood ketone levels during hospitalization.^1^

Diet Group	Admission	Discharge	*t*-value	*p-*value
CON	0.36 ± 0.28	0.30 ± 0.25	0.68	0.5
LCD	0.34 ± 0.41	0.80 ± 0.46	−7.12	<0.001

^1^Data are expressed as mean ± SD (*P*-value from paired samples *t*-test). CON, low-fat control diet group; LCD, low-carbohydrate diet group.

## 4 Discussion

Our study suggests that a short-term LCD intervention significantly improves GV in hospitalized patients with insulin-deficient diabetes, especially those with lower C-peptide levels. Compared to the CON group, patients in the LCD group experienced greater reductions in CV, MAGE, SD, and iAUC, as well as greater improvements in TIR. The LCD also led to reductions in insulin dosage and the number of oral antidiabetic agents used. Importantly, the incidence of hypoglycemia was not increased, and no cases of DKA occurred. This aligns with previous findings that LCDs do not increase the risk of DKA and may even reduce the frequency and duration of hypoglycemic episodes in patients with diabetes, further supporting the short-term safety of this intervention (21–23). In addition, the LCD significantly increased blood ketone levels, which were negatively correlated with blood glucose levels and CV at discharge.

GV has been increasingly recognized as a contributing factor to both microvascular and macrovascular complications in diabetes (24–26). Notably, CV, a validated metric of GV, has been demonstrated to be related to an increased risk of all-cause mortality in T2DM patients (27). Although HbA1c remains the primary clinical target and a well-established predictor of DM complications, GV has gained attention as a complementary metric that may better reflect short-term glucose fluctuations and treatment-associated risks of DKA, hypoglycemia, etc. In this study, we systematically evaluated the short-term effects of an LCD on multiple aspects of GV, including CV, MAGE, SD, TIR, and iAUC, in a large inpatient cohort with confirmed insulin deficiency, thereby contributing valuable insights into the glycemic effects of carbohydrate restriction. Our results demonstrate that short-term LCD intervention significantly improves GV (CV, MAGE, SD, TIR). Since carbohydrate intake is the primary driver of postprandial glucose excursions, lowering carbohydrate consumption directly attenuates GV (28–30). Our previous findings have demonstrated that the lower the carbohydrate intake, the smaller the GV (29). In this context, our findings support the notion that a LCD pattern can effectively stabilize glucose fluctuations. The improved GV in our study is clinically meaningful, as it has been growingly linked to reduced risks of both acute complications of diabetes and long-term vascular outcomes. By demonstrating that LCD markedly reduces GV in insulin-deficient patients, our study highlights an additional therapeutic advantage of carbohydrate restriction beyond HbA1c reduction, underscoring its potential role in optimizing diabetes management.

LCD interventions have been extensively studied in individuals with T2DM, showing benefits in glycemic control and medication reduction (14, 31). However, clinical research in T1DM remains relatively few and is often limited to small-scale studies based on patient self-reported outcomes in free-living settings (32, 33). Notably, a distinct and clinically significant subgroup exists at the intersection of T1DM and advanced T2DM: individuals with severe *β*-cell dysfunction and absolute or near-absolute insulin deficiency. Patients with advanced stages of T2DM, although technically classified as T2DM, exhibit key pathophysiological characteristics of T1DM, including pronounced glycemic variability, strong dependence on exogenous insulin treatment, marked metabolic fragility, and a heightened risk of hypoglycemia and ketoacidosis. Despite their resemblance to T1DM in metabolic profile and clinical management, this insulin-deficient population has received little focused attention in LCD intervention research. The lack of targeted evidence for this group represents a critical gap, particularly given the growing emphasis on individualized diabetes care. Our study directly addresses this gap by thoroughly evaluating the glycemic effects of LCD in a large inpatient cohort with insulin deficiency (fasting C-peptide ≤ 1.0 ng/mL). By focusing on this underrecognized but clinically significant population, we provide insights that are not only relevant to advanced T2DM but also broadly applicable to patients with T1DM-like physiology.

Building upon these primary findings, we further revealed a novel and clinically valuable insight. Patients with lower endogenous insulin secretion, as indicated by lower C-peptide levels (fasting C-peptide < 0.5 ng/mL), exhibited greater improvements in CV following LCD intervention. In other words, individuals with more severely impaired *β*-cell function appeared to derive greater benefit from short-term LCD in terms of glycemic variability improvement. This may be explained by the fact that patients with more impaired β-cell function have a reduced capacity to metabolize dietary carbohydrates. A LCD can therefore alleviate physiological insulin demand by lowering postprandial glucose excursions, making glycemic control less dependent on residual endogenous insulin secretion. In this way, the greater the *β*-cell dysfunction, the more pronounced the benefit derived from carbohydrate restriction in terms of GV improvement. Although previous studies in individuals with diabetes have shown that LCD can improve aspects of glycemic stability, prior investigations have rarely examined heterogeneity of dietary response according to residual β-cell function. To our knowledge, this is the first study to demonstrate such a relationship, emphasizing the potential value of C-peptide-based stratification when considering dietary interventions in insulin-deficient patients of diabetes. This observation highlights β-cell function as a key factor influencing dietary responsiveness and supports a more personalized approach to nutrition therapy in diabetes management.

This study was conducted in a closely monitored inpatient setting with a relatively large sample and detailed continuous glucose monitoring. The controlled hospital environment ensured standardized dietary implementation, insulin titration, and safety surveillance, thereby enhancing the reliability of the observed effects, as well as reducing insulin and oral medication requirements. Specifically, patients in the LCD group showed a 7.2-unit greater net reduction in insulin dosage compared to the CON group. In addition, the LCD group required on average one fewer insulin injection per day at discharge than the CON group, highlighting not only the reduction in dosage but also the simplification of the injection regimen. This strongly suggests that short-term LCD may simplify treatment regimens, lower treatment costs, alleviate the physical and psychological burden of frequent injections, and ultimately improve patient adherence.

Moreover, our study is the first to report that higher circulating ketone levels were consistently associated with both lower blood glucose concentrations and reduced GV. These findings, supported by LMM analyses and clear negative trends across individual patients in scatter plots, suggest that ketone metabolism may play an active role in glucose regulation and glycemic stability. While the underlying mechanisms remain to be clarified, this observation adds to the growing interest in the metabolic effects of ketone bodies and highlights the need for further research in this area.

Several limitations of this study should be acknowledged. First, the non-randomized design may introduce selection bias, as dietary choices were made by patients rather than assigned through random allocation. However, implementing an RCT in this context is challenging, as the acceptance of carbohydrate-restricted diets among patients is strongly age-dependent in our study. In particular, younger patients were generally more willing to adopt LCD, whereas older patients demonstrated resistance to changing long-established dietary habits, which made strict random allocation impractical. This is probably because older adults tend to show greater food neophobia (i.e., reluctance to try novel foods) and stronger reliance on familiar, habitual eating patterns, as well as lower nutrition knowledge and practical barriers (e.g., chewing difficulties), which together reduce their willingness to adopt restrictive or unfamiliar dietary patterns such as LCD (34, 35). Despite baseline imbalances of age and diabetes duration between the CON and LCD groups, simple and stepwise multiple regression analyses demonstrated that these two factors were not associated with GV at discharge. Nevertheless, we performed propensity score matching, and the matched results were consistent with those of the multivariable regression, indicating that the differences in age and diabetes duration did not alter the overall findings. This suggests that the benefits of our short-term LCD intervention were robust and not attributable to baseline confounding. Second, the intervention was conducted in a short-term inpatient setting (median duration 9 days), which ensured high dietary adherence but limited the ability to evaluate long-term efficacy and sustainability of LCD in free-living conditions. In real-world settings, adherence may vary considerably due to factors such as personal food preferences, social eating environments, and the practical challenges of maintaining carbohydrate restriction in daily life. Therefore, the applicability and durability of LCD beyond short-term inpatient care remain uncertain. In addition, while the observed association between ketone levels and CV is noteworthy, it remains uncertain whether the observed association reflects a causal metabolic effect, and thus the finding should be interpreted as exploratory. Third, the interpretation of CGM-derived outcomes is constrained by the small number of participants with analysable discharge CGM data, which limits the robustness of the iAUC comparison. Finally, multiple secondary outcomes (mean, SD, CV, MAGE, TIR, etc.) were analyzed in addition to the prespecified primary endpoint (CV); as these were exploratory analyses, the results should be interpreted with caution. Future randomized controlled trials with longer follow-up are warranted to confirm our findings and further elucidate the mechanistic role of ketone metabolism in glycemic regulation.

## 5 Conclusion

In conclusion, this non-randomized clinical trial demonstrates that a short-term low-carbohydrate diet significantly improves glycemic variability and reduces insulin requirements in hospitalized patients with insulin-deficient diabetes, without increasing the risk of hypoglycemia or ketoacidosis. The benefits were particularly evident in individuals with lower C-peptide levels, suggesting that residual *β*-cell function may modify dietary responsiveness. Moreover, the observed inverse association between blood ketone concentrations and glycemic variability highlights a potential metabolic pathway that may partially contribute to the clinical effects of carbohydrate restriction. While the findings are encouraging, they should be interpreted within the context of the study’s short-term and inpatient design. Nevertheless, our results provide important clinical and mechanistic insights that support the role of personalized nutritional strategies, especially carbohydrate restriction, as a valuable adjunct in the management of insulin-deficient diabetes.

## Data Availability

The original contributions presented in the study are included in the article/Supplementary material, further inquiries can be directed to the corresponding author.

## Glossary

ADA: American Diabetes Association
BMI: Body mass index
CGM: Continuous glucose monitoring
CON: Control diet
CV: Coefficient of variation
CVD: Cardiovascular disease
DBP: Diastolic blood pressure
DKA: Diabetic ketoacidosis
DM: Diabetes mellitus
GV: Glycemic variability
iAUC: Incremental area under the curve
LCD: Low-carbohydrate diet
LMM: Linear mixed model
MAGE: Mean amplitude of glycemic excursions
PSM: Propensity score matching
RR: Relative risk
SBP: Systolic blood pressure
SD: Standard deviation
T1DM: Type 1 diabetes mellitus
T2DM: Type 2 diabetes mellitus
TAR: Time above range
TBR: Time below range
TIR: Time in range

## References

[1] Leslie RD Ma RCW Franks PW Nadeau KJ Pearson ER Redondo MJ. Understanding diabetes heterogeneity: key steps towards precision medicine in diabetes. Lancet Diabetes Endocrinol. (2023) 11:848–60. doi: 10.1016/S2213-8587(23)00159-6, 37804855

[2] Meade LT Rushton WE. Accuracy of carbohydrate counting in adults. Clin Diabetes. (2016) 34:142–7. doi: 10.2337/diaclin.34.3.142, 27621531 PMC5019010

[3] Scott SN Anderson L Morton JP Wagenmakers AJM Riddell MC. Carbohydrate restriction in type 1 diabetes: a realistic therapy for improved Glycaemic control and athletic performance? Nutrients. (2019) 11:22. doi: 10.3390/nu11051022, 31067747 PMC6566372

[4] Famulla S Hövelmann U Fischer A Coester HV Hermanski L Kaltheuner M . Insulin injection into Lipohypertrophic tissue: blunted and more variable insulin absorption and action and impaired postprandial glucose control. Diabetes Care. (2016) 39:1486–92. doi: 10.2337/dc16-0610, 27411698

[5] Hirsch IB. Glycemic variability and diabetes complications: does it matter? Of course it does! Diabetes Care. (2015) 38:1610–4. doi: 10.2337/dc14-2898, 26207054

[6] Ceriello A Monnier L Owens D. Glycaemic variability in diabetes: clinical and therapeutic implications. Lancet Diabetes Endocrinol. (2019) 7:221–30. doi: 10.1016/S2213-8587(18)30136-0, 30115599

[7] Jung HS. Clinical implications of glucose variability: chronic complications of diabetes. Endocrinol Metab. (2015) 30:167–74. doi: 10.3803/EnM.2015.30.2.167, 26194076 PMC4508260

[8] Monnier L Colette C Owens D. The glycemic triumvirate and diabetic complications: is the whole greater than the sum of its component parts? Diabetes Res Clin Pract. (2012) 95:303–11. doi: 10.1016/j.diabres.2011.10.014, 22056719

[9] Newburgh L Marsh PL. The use of a high fat diet in the treatment of diabetes mellitus: first paper. Arch Intern Med. (1920) 26:647–62.

[10] Barber TM Hanson P Kabisch S Pfeiffer AFH Weickert MO. The low-carbohydrate diet: short-term metabolic efficacy versus longer-term limitations. Nutrients. (2021) 13:187. doi: 10.3390/nu13041187, 33916669 PMC8066770

[11] Evert AB Dennison M Gardner CD Garvey WT Lau KHK MacLeod J . Nutrition therapy for adults with diabetes or prediabetes: a consensus report. Diabetes Care. (2019) 42:731–54. doi: 10.2337/dci19-0014, 31000505 PMC7011201

[12] Tay J Luscombe-Marsh ND Thompson CH Noakes M Buckley JD Wittert GA . Comparison of low- and high-carbohydrate diets for type 2 diabetes management: a randomized trial. Am J Clin Nutr. (2015) 102:780–90. doi: 10.3945/ajcn.115.112581, 26224300

[13] Cai L Yin J Ma X Mo Y Li C Lu W . Low-carbohydrate diets lead to greater weight loss and better glucose homeostasis than exercise: a randomized clinical trial. Front Med. (2021) 15:460–71. doi: 10.1007/s11684-021-0861-6, 34185279

[14] Wheatley SD Deakin TA Arjomandkhah NC Hollinrake PB Reeves TE. Low carbohydrate dietary approaches for people with type 2 diabetes-a narrative review. Front Nutr. (2021) 8:687658. doi: 10.3389/fnut.2021.687658, 34336909 PMC8319397

[15] Foster GD Wyatt HR Hill JO McGuckin BG Brill C Mohammed BS . A randomized trial of a low-carbohydrate diet for obesity. N Engl J Med. (2003) 348:2082–90. doi: 10.1056/NEJMoa022207, 12761365

[16] Yunjie G Jing S Jun Y. Clinical study on low-carbon diet for endogenous-insulin-deficient diabetes patients. Chin Gen Pract. (2023) 26:3308–13.

[17] Monnier L Colette C Wojtusciszyn A Dejager S Renard E Molinari N . Toward defining the threshold between low and high glucose variability in diabetes. Diabetes Care. (2017) 40:832–8. doi: 10.2337/dc16-1769, 28039172

[18] Arya P Husain N Kumar C Shekhar R Prakash V Hameed S . C-peptide level in patients with uncontrolled type 2 diabetes mellitus on oral anti-diabetic drugs. Cureus. (2024) 16:e56810 38654804 10.7759/cureus.56810 PMC11036452

[19] Uzunlulu M Oguz A Arslan Bahadir M Erbakan AN Vural Keskinler M Alpaslan MB. C-peptide concentrations in patients with type 2 diabetes treated with insulin. Diabetes Metab Syndr. (2019) 13:3099–104. doi: 10.1016/j.dsx.2019.11.010, 31785503

[20] Ohbatake A Yagi K Karashima S Shima Y Miyamoto Y Asaka H . C-peptide area under the curve at glucagon stimulation test predicts glucose improvements by GLP-1 receptor analogue: a retrospective observational study. Diab. Ther. Res. Treatment Educ. Diabetes Related Disorders. (2019) 10:673–81. doi: 10.1007/s13300-019-0586-6, 30788807 PMC6437227

[21] Nielsen JV Jönsson E Ivarsson A. A low carbohydrate diet in type 1 diabetes: clinical experience--a brief report. Ups J Med Sci. (2005) 110:267–73. doi: 10.3109/2000-1967-074, 16454166

[22] Schmidt S Christensen MB Serifovski N Damm-Frydenberg C Jensen JB Fløyel T . Low versus high carbohydrate diet in type 1 diabetes: a 12-week randomized open-label crossover study. Diabetes Obes Metab. (2019) 21:1680–8. doi: 10.1111/dom.13725, 30924570

[23] Wilmot EG Choudhary P Leelarathna L Baxter M. Glycaemic variability: the under-recognized therapeutic target in type 1 diabetes care. Diabetes Obes Metab. (2019) 21:2599–608. doi: 10.1111/dom.13842, 31364268 PMC6899456

[24] Monnier L Colette C Owens DR. The application of simple metrics in the assessment of glycaemic variability. Diabetes Metab. (2018) 44:313–9. doi: 10.1016/j.diabet.2018.02.008, 29602622

[25] Frontoni S Di Bartolo P Avogaro A Bosi E Paolisso G Ceriello A. Glucose variability: an emerging target for the treatment of diabetes mellitus. Diabetes Res Clin Pract. (2013) 102:86–95. doi: 10.1016/j.diabres.2013.09.007, 24128999

[26] Lu J Ma X Zhou J Zhang L Mo Y Ying L . Association of Time in range, as assessed by continuous glucose monitoring, with diabetic retinopathy in type 2 diabetes. Diabetes Care. (2018) 41:2370–6. doi: 10.2337/dc18-1131, 30201847

[27] Mo Y Wang C Lu J Shen Y Chen L Zhang L . Impact of short-term glycemic variability on risk of all-cause mortality in type 2 diabetes patients with well-controlled glucose profile by continuous glucose monitoring: a prospective cohort study. Diabetes Res Clin Pract. (2022) 189:109940. doi: 10.1016/j.diabres.2022.109940, 35662611

[28] Song J Oh TJ Song Y. Individual postprandial glycemic responses to meal types by different carbohydrate levels and their associations with glycemic variability using continuous glucose monitoring. Nutrients. (2023) 15:3571. doi: 10.3390/nu15163571, 37630761 PMC10459284

[29] Cai L Xia X Gu Y Hu L Li C Ma X . Opposite effects of low-carbohydrate high-fat diet on metabolism in humans and mice. Lipids Health Dis. (2023) 22:191. doi: 10.1186/s12944-023-01956-3, 37950240 PMC10636972

[30] Chang CR Francois ME Little JP. Restricting carbohydrates at breakfast is sufficient to reduce 24-hour exposure to postprandial hyperglycemia and improve glycemic variability. Am J Clin Nutr. (2019) 109:1302–9. doi: 10.1093/ajcn/nqy261, 30968140 PMC6499564

[31] Goldenberg JZ Day A Brinkworth GD Sato J Yamada S Jönsson T . Efficacy and safety of low and very low carbohydrate diets for type 2 diabetes remission: systematic review and meta-analysis of published and unpublished randomized trial data. BMJ. (2021) 372:m4743. doi: 10.1136/bmj.m4743, 33441384 PMC7804828

[32] Neuman V Plachy L Drnkova L Pruhova S Kolouskova S Obermannova B . Low-carbohydrate diet in children and young people with type 1 diabetes: a randomized controlled trial with cross-over design. Diabetes Res Clin Pract. (2024) 217:111844. doi: 10.1016/j.diabres.2024.111844, 39237039

[33] Levran N Levek N Sher B Gruber N Afek A Monsonego-Ornan E . The impact of a low-carbohydrate diet on micronutrient intake and status in adolescents with type 1 diabetes. Nutrients. (2023) 15:418. doi: 10.3390/nu15061418, 36986149 PMC10051868

[34] Linschooten JO Verwijs MH Beelen J MAE d v d S Roodenburg AJC. Low awareness of community-dwelling older adults on the importance of dietary protein: new insights from four qualitative studies. J Nutr Sci. (2021) 10:e102 35059183 10.1017/jns.2021.92 PMC8727701

[35] van den Heuvel E Newbury A Appleton KM. The psychology of nutrition with advancing age: focus on food Neophobia. Nutrients. (2019) 11:151. doi: 10.3390/nu11010151, 30642027 PMC6356997

