# Supplementary material for: The beneficial impact of a low-carbohydrate diet on glycemic variability in insulin-deficient diabetes
Source: Front Nutr. 2026 Jan 9;12:1733037. doi: 10.3389/fnut.2025.1733037 (PMC12827080; doi:10.3389/fnut.2025.1733037)
Supplement: Supplementary file 1 [file Table_1.docx]

Supplementary Table 1. Baseline characteristics of study participants^1^

|  | CON | LCD |  |  |
| --- | --- | --- | --- | --- |
| Characteristics | *n* = 105 | *n* = 105 | Statistics | *P* value |
| Age, years | 61.9 ± 7.7 | 60.4 ± 10.1 | 1.3 | 0.2 |
| Male Gender, n(%) | 59(56.2%) | 55(52.4%) | 0.2 | 0.7 |
| Duration, years | 10.0 [3.0 ~ 17.0] | 9 [1.0 ~ 15.0] | 1.3 | 0.2 |
| SBP, mmHg | 131.4 ± 17.8 | 131.3 ± 17.7 | 0.03 | 0.98 |
| DBP, mmHg | 77.3 ± 9.8 | 78.2 ± 10.3 | -0.7 | 0.5 |
| BMI, kg/m2 | 23.0 [20.7 ~ 24.4] | 23.1 [20.9 ~ 24.9] | -0.8 | 0.4 |
| C-peptide, ng/ml | 0.62 [0.41 ~ 0.84] | 0.63 [0.39 ~ 0.83] | 0.1 | 0.9 |
| HbA1c, % | 10.5 [8.9 ~ 12.6] | 10.3 [8.5 ~ 12.2] | 1 | 0.3 |
| CV(%) | 37.24 ± 11.88 | 37.03 ± 12.28 | 0.1 | 0.9 |
| MAGE | 7.46 ± 3.28 | 7.52 ± 3.92 | -0.1 | 0.9 |
| SD | 3.98 ± 1.48 | 3.94 ± 1.68 | 0.2 | 0.9 |
| Mean | 10.76 ± 2.45 | 10.59 ± 2.53 | 0.5 | 0.6 |
| TIR (%) | 51.47 ± 25.46 | 55.22 ± 24.67 | -1.1 | 0.3 |
| TAR (%) | 46.63 ± 25.36 | 42.98 ± 25.11 | 1.1 | 0.3 |
| TBR (%) | 1.9 ± 4.75 | 1.81 ± 5.46 | 0.1 | 0.9 |

^1^Data are expressed as mean ± SD, median (interquartile range) or n (%).

Abbreviation: CON, low-fat control diet group; LCD, low-carbohydrate diet group, SBP, systolic blood pressure; DBP, diastolic blood pressure; BMI, body mass index; HbA1c, glycated hemoglobin A1c; GA, glycated albumin.

Supplementary Table 2. Comparison of dietary effects on improvements in glycaemic variability across the intervention period^1^

|  | Admission | | Discharge | | Relative LC Effect Across Intervention | *P* Value^2^ |
| --- | --- | --- | --- | --- | --- | --- |
|  | CON | LCD | CON | LCD |  |  |
| CV(%) | 37.2 ± 11.9 | 37.0 ± 12.3 | 28.0 ± 10.4 | 21.1 ± 7.9 | -6.7 (-10.5~-3.0) | <0.01 |
| MAGE | 7.5 ± 3.3 | 7.5 ± 3.9 | 5.0 ± 2.5 | 3.1 ± 1.7 | -1.8 (-2.8~-0.9) | <0.01 |
| SD | 4.0 ± 1.5 | 3.9 ± 1.7 | 2.5 ± 1.2 | 1.7 ± 0.7 | -0.8 (-1.2~-0.4) | <0.01 |
| Mean | 10.8 ± 2.5 | 10.6 ± 2.5 | 8.7 ± 1.5 | 7.9 ± 1.2 | -0.6 (-1.4~1.) | 0.1 |
| TIR (%)^3^ | 51.5 ± 25.5 | 55.2 ± 24.7 | 76.1 ± 19.2 | 86.8 ± 16.8 | 0.6 (0.2~1.0) | <0.01 |
| TAR (%)^3^ | 46.6 ± 25.4 | 43.0 ± 25.1 | 23.0 ± 18.9 | 12.3 ± 16.7 | -0.7 (-1.1~-0.2) | <0.01 |
| TBR (%)^3^ | 1.9 ± 4.7 | 1.8 ± 5.5 | 0.9 ± 3.2 | 0.8 ± 3.4 | 0.01 (-0.37~0.39) | 0.9 |

^1^Data by group are presented as mean ± SD, relative effect of LC diet across intervention is presented as effect estimates (95% CI).

^2^Data analyzed via linear mixed model with fixed effects for timepoints (Admission compared with Discharge), dietary intervention group, and the interaction between timepoint and dietary intervention group, and a random effect for participant.

^3^Data were converted to and are presented as proportion of time in range and analyzed via mixed effects beta model. Interpret effect estimates as % relative change in odds.

Abbreviation: CON, low-fat control diet group; LCD, low-carbohydrate diet group; CV, coefficient of variation; MAGE, mean amplitude of glycemic excursions; SD, standard deviation; TIR, time between 3.9 and 10 mmol/L; TAR, time above 10 mmol/L; TBR, time below 3.9 mmol/L

Supplementary Table 3. Comparison of daily insulin dosage and oral anti-diabetic agents between different dietary intervention at admission and discharge

|  | CON | LCD | *P* value |
| --- | --- | --- | --- |
| Daily insulin dosage^1^ |  |  |  |
| Admission | 20.3 ± 14.8 | 16.9 ± 12.1 | 0.06 |
| Discharge | 24.9 ± 13.9 | 13.0 ± 7.3 | <0.001 |
| Insulin injection frequency  at discharge (per day)^2^ | 2 [1 ~ 4] | 1 [1 ~ 1] | <0.001 |
| Types of oral anti-diabetic agents^3^  (0/1/2/3 types) |  |  |  |
| Admission | (83/19/3/0) | (90/15/0/0) | 0.18 |
| Discharge | (16/48/39/2) | (77/25/3/0) | <0.001 |

^1^Data are expressed as mean ± SD (*P* value from independent samples t test).

^2^Data are expressed as median (interquartile range) (*P* value from Mann–Whitney U test).

^3^Data are expressed as (n_0_/n_1_/n_2_/n_3_) (*P* value from Fisher's exact test).

Abbreviation: CON, low-fat control diet group; LCD, low-carbohydrate diet group.

Supplementary Table 4. Dietary intervention and low discharge CV (discharge CV <36%) in all participants, stratified by C-peptide levels^1^

| C-peptide levels | CON | | LCD | | RR(95CI%) | *P*  value |
| --- | --- | --- | --- | --- | --- | --- |
|  | n | discharge cv＜36%, n(%) | n | discharge cv＜36%, n(%) |  |  |
| < 0.5 ng/ml | 40 | 25(62.5%) | 35 | 33(94.3%) | 1.5(1.2-1.9) | 0.001 |
| ≥ 0.5 ng/ml | 65 | 60(92.3%) | 70 | 65(92.9%) | 1.0(0.9-1.1) | 0.9 |
| Test for  interaction^2^ |  |  |  |  |  | 0.003 |

^1^Data analyzed via generalized linear model adjusted for baseline glycaemic variability (admission CV).

^2^*P* value for interaction test: 2-way interaction of diet (Low-Carbohydrate vs. Normal Diet) and C-peptide levels (<0.5 vs. ≥0.5) on discharge CV.

Abbreviation: CON, low-fat control diet group; LCD, low-carbohydrate diet group; RR, relative risk; CI, confidence interval.

Supplementary Table 5. Impact of reference diet and LC diet on blood ketone levels during hospitalization^1^

| Diet Group | Admission | Discharge | *t* value | *P* value |
| --- | --- | --- | --- | --- |
| REF | 0.40±0.71 | 0.32±0.30 | 0.6 | 0.6 |
| LC | 0.31±0.23 | 0.81±0.46 | -8.3 | <0.001 |

^1^Data are expressed as mean ± SD (*P* value from paired samples *t* test).

Abbreviation: CON, low-fat control diet group; LC, low-carbohydrate diet group

Supplementary Table 6. ANCOVA of discharge CV adjusting for baseline CV^1^

| Variable | Estimate | 95% CI | *P* value |
| --- | --- | --- | --- |
| CV (discharge) | -4.79 | -6.66 ~ -2.92 | <0.001 |

^1^Data analyzed via ANCOVA, with discharge CV as the dependent variable and baseline CV included as a covariate. The estimate represents the adjusted between-group difference in discharge CV (CON vs. LCD).

Abbreviation: ANCOVA, analysis of covariance; CI, confidence interval

Supplementary Table 7. Percent change in total daily insulin dose from admission to discharge between the two dietary groups^1^

| Variable | CON (*n* = 171) | LCD (*n* = 96) | *t* value | *P* value |
| --- | --- | --- | --- | --- |
| Percent change in insulin dose (%) | -8.4 ± 51.2 | -33.8 ± 41.9 | 4.38 | <0.001 |

^1^Data are expressed as mean ± SD (*P* value from independent samples *t* test).

Abbreviation: CON, low-fat control diet group; LCD, low-carbohydrate diet group.
